# Tools for the Assessment of Pediatric Restless Legs Syndrome

**DOI:** 10.3389/fpsyt.2020.00356

**Published:** 2020-05-05

**Authors:** Pamela Hamilton Stubbs, Arthur S. Walters

**Affiliations:** ^1^Dr. Hamilton-Stubbs Sleep and Total Wellness Institute, LLC., Richmond, VA, United States; ^2^Sleep Division, Department of Neurology, Vanderbilt University School of Medicine, Nashville, TN, United States

**Keywords:** restless legs syndrome (RLS), diagnosis, severity, quality of life, sleep, pediatric, childhood, adolescence

## Abstract

**Background:**

There are only a few of the questionnaires for the diagnosis, severity and quality of life of adult Restless Legs Syndrome (RLS) that have been utilized in children. Even fewer of these types of instruments have been developed specifically for Pediatric RLS.

**Methods:**

This article is a review of instruments used in adult RLS, their applicability to children and of instruments specifically developed for childhood RLS.

**Results:**

A single question for the diagnosis of RLS has been validated for adults and utilized in one epidemiology study of adolescents with RLS. The Pediatric Emory RLS questionnaire has been developed as a diagnostic instrument for childhood RLS, utilized in two studies of RLS in children, but not yet validated. The IRLS (International Restless Legs Scale), the CGI (Clinical Global Impression), and the RLS-6, which have been validated for determining adult RLS severity, were administered without difficulty in one therapeutic study of adolescent RLS. In addition, the IRLS has also been utilized in another 5 studies of childhood and adolescent RLS. The pediatric Restless Legs Syndrome Severity Scale (P-RLS-SS) has been developed for use in children but not yet validated. A modification of the P-RLS-SS based upon rating the severity of the 4 diagnostic criteria for RLS has been developed for children but not yet validated. There are no Quality of Life scales developed for Pediatric RLS. However, 3 separate studies utilized the general Peds Quality of Life Inventory (PedsQL) in RLS children and adolescents and one of these studies also employed the general Sleep Behavior Questionnaire (SBQ) and yet another of these studies also employed the Pediatric Symptom checklist (PSC).

**Discussion:**

There is a need for the development and validation of instruments specific to Pediatric RLS. Meanwhile, we recommend the use of the Pediatric RLS instruments that have been developed and we recommend use of the adult scales in adolescent RLS where language barriers are not a problem. If adult scales are used in younger children, we recommend that they be administered in conjunction with an ongoing discussion between the parent and the child during the scale administration.

## Introduction

Restless legs is a common disorder in adults and children. Approximately 25% of adults with RLS report symptoms onset between the ages of 10–20 years. Restless legs syndrome is known to adversely affect quality of life. The full impact of RLS upon children is unknown. Children with RLS have an increased incidence of co-morbid conditions such as attention deficit disorder, iron deficiency anemia, and parasomnias (1–4).

There are only a few of the questionnaires for the diagnosis, severity, and quality of life of adult restless legs syndrome (RLS) that have utilized in children. Even fewer of these types of instruments have been developed specifically for pediatric RLS. This article is a review of instruments used in adult RLS, their applicability to children and of instruments specifically developed for childhood RLS.

The American Academy of Sleep Medicine through the International Classification of Sleep Disorders 3 (ICSD3), the American Psychiatry Association through the Diagnostic and Statistical Manual of Mental Disorders 5 (DSM 5), and International Restless Legs Syndrome Study Group (IRLSSG) have each developed separate diagnostic criteria for RLS (1–4). The three sets of criteria have minor differences. However, all three sets of criteria require the presence of the same five essential features (1–4).

1. Urge to move the legs usually due to abnormal sensations
2. The worsening or onset of symptoms associated with rest or inactivity
3. Temporary relief or partial relief of symptoms by movement, walking, or stretching
4. Symptoms are more severe later in the day, in the evening or night
5. Mimics of RLS such as leg cramps, positional discomfort, myalgia, venous stasis, leg edema, arthritis, habitual foot tapping are to be excluded. Mimics are those disorders that meet all or almost all of the primary criteria for RLS but are not RLS.

In addition, and most importantly for the diagnosis of definite childhood RLS, all three sets of criteria indicate that the diagnosis of childhood RLS requires that the children must be able to describe the RLS symptoms in their own words.

Frequency of symptoms and duration are not included in all three sets of criteria. The IRLSSG divides duration into chronic persistent (at least two times per week for the past year) and intermittent (less than two times per week for the past year with at least five lifetime episodes). The DSM-5 requires a frequency of three times per week for at least 3 months. The ICSD-3 does not specify frequency or duration. Unlike the DSM-5 criteria the IRLSSG and ICSD3 criteria allow the diagnosis of milder and intermittent forms of RLS (**Table 1**) (1–4).

**Table 1:** Criteria for restless legs syndrome (RLS) by different organizations.

Feature	IRLSSG	ICSD-3	DSM-5
5 essential features	Same	Same	Same
Frequency	Chronic persistent or Intermittent*	NA	3 times per week
Duration	Persistent or intermittent over 1 year	NA	3 months
Clinical significance	Can cause impairment but impairment is not required	Requires impairment with exception of genetic and epidemiological studies	Requires impairment
Waivers	Frequency and duration may be waivered in childhood, pregnancy, and drug induced RLS	Impairment requirement can be waived in genetic and epidemiological studies	NA
Recognizes drug induced RLS	Yes	Yes	No
RLS mimics must be excluded	Yes	Yes	Yes
Children/adolescents must describe symptoms in own words	Required	Required	Required

^*^See full definition of chronic persistent and intermittent in text.

In 2013 the executive committee of the IRLSSG appointed a task force to update pediatric RLS diagnostic criteria (4). Simplified pediatric criteria were developed with specific recommendations for application in pediatric populations. The updated pediatric RLS diagnostic criteria are expected to improve clinical practice and facilitate research (1–4). For the purpose of this review, we used the IRLSSG consensus diagnostic criteria for RLS (2–4). The Pediatric RLS Severity Scale (P-RLS-SS) also developed through a subcommittee of the IRLSSG in conjunction with industry is a self-administered survey of symptoms and assessment of impact of RLS upon four domains (5).

### The International Restless Legs Syndrome Study Group Consensus Diagnostic Criteria for Restless Legs Syndrome

1. A desire to move one or both legs that may or may not be accompanied by or caused by discomfort and unpleasant sensations in the legs.
2. Periods of rest or inactivity precipitate or worsen the desire to move the legs and associated unpleasant sensations.
3. Symptoms are reduced or completely eliminated by movement or symptoms are reduced or eliminated for the duration of movement.
4. Symptoms are more severe or limited to evening or night.
5. Symptoms cannot be solely caused by another medical or behavioral condition.

#### Specifier for Clinical Significance of Restless Legs Syndrome in Adults

Social, occupational, educational, or other important areas of functioning are adversely affected by the impact of RLS on sleep, energy/vitality, daily activities, behavior, cognition, or mood (2).

### Diagnosis of Restless Legs Syndrome in Children

To promote accuracy, consistency in diagnosis, in 2013 the pediatric diagnostic criteria were updated and are the same as diagnostic criteria for adults with special considerations (2, 4):

1. The child must report RLS symptoms in his or her own words.
2. The diagnostician should be aware of the vocabulary children and adolescents may use to describe symptoms.
3. Application of RLS diagnostic criteria are determined by language and cognitive development and not age.
4. It is not known if the adult specifiers for clinical course apply to pediatric RLS.
5. As in adults, a significant impact on sleep, mood cognition, and function is found. Impairment is manifest more often in behavioral and educational domains.
6. Simplified and updated research criteria for probable and possible pediatric RLS are available.
7. Periodic limb movement disorder may precede the diagnosis of RLS in some cases.

### Specifiers for Clinical Course of Restless Legs Syndrome in Adults and Children

- Chronic-persistent RLS: without treatment, symptoms occur a minimum average of twice per week for 1 year
- Intermittent RLS: without treatment symptoms are present on average <2/week for the past year and there is a history of at least five lifetime events

The diagnosis of RLS is based on the medical evaluation. A structured validated assessment tool can aid an accurate clinical diagnosis and facilitates research in assessment and diagnosis of RLS (2–4).

## Method

A literature search was completed to identify screening tools used to diagnose RLS, rate the severity of RLS symptoms, and tools to evaluate the impact of RLS upon quality of life. Each tool was reviewed for applicable use in the evaluation of RLS in pediatric populations.

### Results

Assessment tools were assigned to one of four categories:

1. Diagnostic tools
2. Severity scales
3. Quality of life scales
4. Quality of life instruments specific to sleep

A summary of the results and applicability to children is provided in **Table 2**.

**Table 2:** Summary of assessment tools for restless legs syndrome.

Assessment tool	Validated in adults	Utilized in children
**Cambridge-Hopkins Diagnostic Questionnaire (CH-RLSq)**	Yes	No
**Hening telephone diagnostic interview (HTDI)**	Yes	No
**RLS Diagnostic Index**	Yes	No
**Single question for RLS**	Yes	Yes
**The RLS-Expanded Questionnaire**	Yes	No
**The AIIMS RLS questionnaire for Indian patients (ARQIP)**.	Yes	No
**Pediatric Emory RLS diagnostic questionnaire**	No	Yes
**The RLSQ**	No	Yes
**International Restless Legs Scale (IRLS)**	Yes	Yes
**Self-administered version of the International Restless Legs Syndrome study group severity rating scale (sIRLS)**.	Yes	No
**Clinical Global Impressions Rating Scales (CGI)**	Yes	Yes
**RLS-6 scale of restless legs syndrome/Willis-Ekbom disease**	Yes	Yes
**Johns Hopkins Restless Legs Severity Scale (JHRLSS)**	Yes	No
**Augmentation Severity Rating Scale (ASRS)**	Yes	No
**Pediatric Restless Legs Syndrome Severity Scale (P-RLS-SS)**	No	Yes
**Rating the four RLS diagnostic criteria**	No	Yes
**Kohnen RLS quality of life instrument**	Yes	No
**The RLS Quality of Life Questionnaire—Abetz**	Yes	No
**The Restless Legs Syndrome Quality of Life Instrument (RLS-QLI)**	Yes	No
**The post-sleep questionnaire for RLS (PSQ)**	Yes	No
**The RLS next day impact questionnaire**	Yes	No
**The subjective post-sleep diary (SPSD) for RLS**	Yes	No

Note from the authors: There are no assessment tools validated in pediatric populations.

## Results

### Diagnostic Tools for Adult Restless Legs Syndrome

#### Cambridge-Hopkins Diagnostic Questionnaire

The Cambridge-Hopkins diagnostic questionnaire (CH-RLSq) is a self-administered questionnaire developed for large scale epidemiological studies (6). It does not require a diagnostician to be present. Questions cover the essential diagnostic criteria and the two most common RLS mimickers, leg cramps, and positional leg discomfort. The instrument allows patients to be categorized into one of three groups: has RLS, does not have RLS, and probable RLS.

The CH-RLSq is a validated patient completed questionnaire capable of identifying patients likely to have RLS. The scale was validated against the Hening telephone diagnostic interview (HTDI) (see below) which served as the “gold standard.” The CH-RLSq has a sensitivity of 87.2% and specificity of 94.4%. The positive predictive value is 85.5% but researchers state the high positive predictive value may be secondary to the high risk of RLS within the test population who were blood donors. The researchers estimate a positive predictive value of 63.4% in a general population (6).

The CH-RLSq is used to screen populations for RLS. If positive the HTDI can be used to confirm RLS. However, the CH-RLSq can also be used as a stand-alone assessment tool. To our knowledge the CH-RLq has not been applied as a stand-alone questionnaire in children.

#### Hening Telephone Diagnostic Interview

The Hening telephone diagnostic interview (HTDI) consists of questions based on the diagnostic criteria for RLS established by the IRLSSG as well as questions to exclude leg cramps (7). It is not a patient completed instrument. It was developed for the diagnostician to complete during a telephone interview with the patient but has been used during face to face interviews. The HTDI allows the diagnostician to place the patient into one of three categories: definite RLS, probable RLS, or does not have RLS. The updated version of the HDTI includes questions to help differentiate positional leg discomfort, another common RLS mimic. The revised version, to the best of our knowledge, remains to be validated.

The “gold standard” for diagnosing RLS is the clinical interview. The HTDI was validated by having patients undergo dual interviews by clinical experts in RLS. The rate of agreement between dual clinicians was 93–96%. The HTDI has a specificity of 91%, a sensitivity of 90%, a positive predictive value of 86–89%, and a negative predictive value of 94% (7).

The HTDI was developed for telephone interviews. In our opinion the HTDI would be a reliable tool for telemedicine interviews. The current IRLSSG guidelines require children to describe symptoms in their own words. The HTDI does not include descriptive language used by young children and to our knowledge the HTDI has not been applied to children.

#### Restless Legs Syndrome Diagnostic Index

This was developed to take into account other non-essential features of RLS that, if taken into account, might improve the diagnostic accuracy of an interview for RLS (8). There are 10 items and 5 address the essential features of RLS and 5 the non-essential features. These non-essential but frequently found features include sleep disturbance, a positive family history of RLS, the bettering of RLS symptoms with dopaminergic therapy, and the presence of periodic limb movements in sleep (PLMS) on an overnight sleep study as well as whether the RLS can be explained by a co-morbid medical condition (8). The RLS-DI has been validated in adults against the diagnosis of two independent sleep experts but to our knowledge this instrument has not been used in children. In adults the sensitivity was 93%, specificity was 98.9%, positive predictive value 98.8%, negative predictive value 93.9%, and 96.1% of the subjects could be identified correctly.

#### Single Question for Restless Legs Syndrome

The single question incorporates the NIH criteria for RLS: “when you try to relax in the evening or sleep at night, do you ever have unpleasant, restless feelings in your legs that can be relieved by walking or movement?” (9). The single question has been validated in adults against an in-person interview by two clinicians and has been used in at least once epidemiology study of RLS in adolescents (10). The single question shows a sensitivity of 100%, specificity of 96.8%, positive predictive value of 89.6%, and negative predictive value of 100%.

#### The Restless Legs Syndrome-Expanded Questionnaire

In this study the authors developed two questionnaires for the diagnosis of RLS and validated them both against an in person examination by two RLS experts which served as the gold standard (11). The first questionnaire was based upon the four diagnostic criteria for RLS and the second questionnaire was also based upon the four diagnostic criteria but added additional questions to eliminate mimics such as leg cramps as well as questions regarding sleep disturbance and other demographic information. To our knowledge the RLS-Expanded Questionnaire has not been applied as a stand-alone questionnaire for children. In adults the RLS-Expanded Questionnaire has a sensitivity of 81% and a specificity of 73%.

#### The All India Institute of Medical Sciences Restless Legs Syndrome Questionnaire for Indian Patients

The All India Institute of Medical Sciences (AIIMS) RLS questionnaire for Indian patients (ARQIP) is an expanded physician administered detailed questionnaire developed to uncover salient features required to diagnose RLS (12). All IRLSSG diagnostic criteria plus additional socio-cultural questions appear on the ARQIP. Socio-cultural questions were added to address aspects specific to Hindi culture, the use of culture specific terminology to describe symptoms, and the factors that alleviate symptoms. The questionnaire was developed in English and translated to Hindi.

The ARQIP was compared to the four diagnostic criteria for RLS. The evaluation procedure used two different examiners to administer one questionnaire, either the ARQIP or the four diagnostic criteria for RLS. Questionnaires were administered by face to face interview or telephone interview. All patients were given a final evaluation by an RLS expert who diagnosed each patient as having RLS or “no-RLS” which was considered the “gold standard” for the diagnosis. The ARQIP was was found to have greater sensitivity (100 *vs.* 73%), specificity (44 *vs.* 32.7%), negative predictive value (100 *vs.* 36.4%), and positive predictive value (79 *vs.* 70%) than the IRLSSG criteria administered alone (12). The ARQIP has been used to study RLS in adults (12). We did not find pediatric studies that used the ARQIP.

### Diagnostic Tools Specific to Pediatric Restless Legs Syndrome

#### Pediatric Emory Restless Legs Syndrome Diagnostic Questionnaire

The pediatric Emory RLS diagnostic questionnaire is based on National Institute of Health consensus guidelines for pediatric RLS diagnostic criteria and questions adapted from the Cambridge-Hopkins diagnostic questionnaire (CH-RLSq), the RLS-Expanded Questionnaire and the Phenotypic Presentation of Restless Legs Syndrome Questionnaire (13). Questions are modified for use in pediatric populations.

The questionnaire consists of two sets of similar questions developed for two different age groups: 8–12 years old and 13–18 years old. For children less than 13 years of age, the questionnaire is completed by the parent or primary caretaker in the presence of the child. For the younger children there are 47 questions. For the older children there are 46 questions. The first five questions of the instrument are screening questions.

1. The presence of growing pains
2. Difficulties sitting or lying still
3. An urge or strong need to move the legs due to uncomfortable feelings or sensations
4. A recurrent need to move the legs while sitting or lying down
5. History of leg rubbing or massage to relieve sensations

If any screening question is answered affirmatively, a series of age-specific questionnaires to evaluate details of the discomfort or sensations follow. The questionnaire addresses medications, RLS mimics, and quality of sleep. Both questionnaires provide front and back human images where the affected body part can be shaded. Questions 34–47 for children 8–12 years of age and questions 33–46 for adolescents focus on sleep disturbance. Both sets of questionnaires provide instructions for the classification of definite RLS, probable RLS, and the presence of sleep disturbance.

The pediatric Emory RLS diagnostic questionnaire has been used in a study to evaluate pediatric patients with chronic kidney disease (13) and in a separate study in children with nephrotic syndrome (14). The authors later noted one limitation of the pediatric Emory RLS diagnostic questionnaire is that the questionnaire has not been fully validated. The scale is published in its entirely as an appendix to the aforementioned article on pediatric RLS in chronic kidney disease (13).

#### The Restless Legs Syndrome Questionnaire

The RLSQ is a parent report questionnaire developed for the identification of pediatric RLS by a triangulation process of literature review, parent interviews, and a children’s focus group (15). The final questionnaire has 11 items. The authors state that the questionnaire has been validated and is reliable with an internal consistency of 65% and repeat measure reliability rho = 0.58. However, the details of the validation process were not stated in the article and the scale itself was not published with the original article.

### Severity Scales for Adult Restless Legs Syndrome

#### International Restless Legs Scale

The IRLS is a 10-question scale administered to the patient by an examiner but the patient does the severity rating. The examiner is available to answer any questions the patient has about the items (16). Severity is rated on each question as follows: 0= no symptoms, 1–mild, 2–moderate, 3–severe, and 4–very severe for a total score of 0–40. The IRLS has a severity subscale and an impact on quality of life subscale. The IRLS was originally validated correlating the total score to the Clinical Global Impression (CGI) (r = 0.74) and Patient Global Impression (r = 0.82) and readily distinguished patients from controls (16). The IRLS has been employed in at least two therapeutic studies in adolescents with RLS 13–18 years of age (17) or children with RLS aged 5–12 years (18), in two quality of life studies of children and adolescents aged 7–18 (19) and 12–20 (20), respectively, and in a study of the prevalence of RLS in children and adolescents with allergic rhinitis aged 8–18 years (21) and another study of the prevalence of RLS in children and adolescents with celiac disease aged 11–18 years (22). In one of these studies it is carefully explained that the scale was administered after a thorough discussion of symptoms and daytime function with the children and their parents (19). However, it is to be emphasized that the adult scale has not been validated in children or adolescents and if it is to be administered in these groups, we would recommend that it be done with the cautions above. The IRLS is the most utilized of the severity scales and is employed in most academic and pharmaceutically based studies of RLS therapy. The scale is copyrighted by the International Restless Legs Syndrome Study Group (IRLSSG) with the Mapi Research Trust as the official licensor and distributor. For use contact https://eprovide.mapi-trust.org/. For individual clinical or research use the scale is available at a nominal cost. The scale has undergone minor revisions since its original validation for better readability and the recommendation that the scale be rated for symptoms over the past 1 week rather than the past 2 weeks. Please contact Mapi for the latest version.

#### Self-Administered Version of the International Restless Legs Syndrome Study Group Severity Rating Scale

The first version of the IRLS (see above) was validated under conditions where the examiner had to be present with the patient in order to clarify any misunderstandings the patient might have about the questions that comprise the scale. The second version of the scale (sIRLS) was validated under conditions where the patient did not have to have the examiner present or available (23). The validation study which compared both types of administration indicated that the sIRLS was reliably answered when administered without the presence of the examiner and could theoretically be employed in mass mailings that would reach much larger numbers of patients. The correlation between the IRLS and the sIRLS was 0.94. This scale has not been applied to children but, by extension, the successful use of the IRLS in adolescents would be expected to be true for the sIRLS as well. The scale is copyrighted by the International Restless Legs Syndrome Study Group (IRLSSG) with the Mapi Research Trust as the official licensor and distributor. For use contact https://eprovide.mapi-trust.org/. For individual clinical or research use the scale is available at a nominal cost.

#### Clinical Global Impressions Rating Scales

Clinical Global Impressions of change are a standard tool used in severity assessment for many conditions and is not specific to restless legs syndrome. The CGI can be administered to the patient to get the patient’s impression of the severity of their condition or it can be administered by the treating physician to get the treating physician’s impression of the severity of the patient’s condition. It has been employed in a study specific to adolescents with RLS in at least one instance (17). It is the most frequently employed tool to accompany the IRLS in therapeutic trails of medications for RLS.

#### Restless Legs Syndrome-6 Scale of Restless Legs Syndrome/Willis-Ekbom Disease

The RLS-6 has six items rated from 0 to 10 where the symptoms are rated over the past week (24). The items include severity of RLS at falling asleep, during the night, during the day when sitting or lying, and during the day when active. Another item probes daytime sleepiness and yet another asks how satisfied patients were with their sleep over the last seven nights. The scale has been validated in adults against the IRLS (see above) which served as the gold standard and has been employed in at least one therapeutic study of RLS in adolescents (17). The correlation coefficients of the RLS-6 items ranged from 0.35 to 0.67 with the IRLS total score. The European Restless Legs Syndrome Study Group (EURLSSG) owns intellectual property rights over the RLS-6 with Mapi Research Trust assigned for the management of instrument license and permission to use. For use please consult the Mapi Research Trust website http://www.proqolid.org. For individual clinicians and investigators the scale is available at a nominal fee.

#### Johns Hopkins Restless Legs Severity Scale

This consists of a single question that rates the usual time of day for onset of RLS for at least 50% of days (25). The symptoms are rated: 0= no symptoms; 1 = bedtime symptoms after or within an hour of going to bed; 2= evening and bedtime symptoms starting at or after 6:00 PM; 3 =day and night symptoms starting before 6:00 PM. The scale has been validated in adults against polysomnographic parameters such as periodic leg movements of sleep (R = 0.45, P=0.01) and sleep efficiency (R=0.60; P < .01) but to our knowledge has not been employed in children.

#### Augmentation Severity Rating Scale

In restless legs syndrome treatment with dopaminergic agonists may dramatically improve symptoms at night but may have a side effect of pushing the symptoms into the daytime (26). A generalized paradoxical worsening of RLS symptoms with, for example, spread to other body parts beyond the legs may also occur. This phenomenon is called Augmentation and the Augmentation Severity Rating Scale (ASRS) was designed to measure this phenomenon (26). The scale has three items evaluated over the previous week. The first item asks the time of day the symptoms began over the past week; the second item asks how quickly symptoms developed when sitting at various times of the day in the past week; the third item asks what body parts were involved over the past week. The instrument has been validated in adults against the independent opinion of two clinical experts and the correlation between the worst ASRS total score and expert rating was 0.72. To our knowledge the ASRS has not been employed in children.

### Severity Scales Specific for Pediatric Restless Legs Syndrome

#### Pediatric Restless Legs Syndrome Severity Scale

The Pediatric Restless Legs Syndrome Severity Scale (P-RLS-SS) is a Likert-type 41 item questionnaire based upon interviews of children and adolescents 6–17 years of age conducted at multiple centers (5). The P-RLS-SS was developed using concept elicitation interviews; generation of questions based on conceptual frameworks for measuring symptoms and impact of RLS; cognitive debriefing interviews of children and parents and qualitative analysis of the data using ATLAS.ti software and grounded-theory methods.

Development of the P-RLS was supervised by an advisory board of P-RLS experts and developers of the adult RLS Severity Scale. The P-RLS-SS measures symptoms commonly associated with RLS and the impact of RLS upon four domains: sleep, awake activities, emotions, and tiredness. A separate complementary parent questionnaires allows parents to provide observations regarding mood changes and information the child may not be aware of, such as sleeping in unusual positions.

The P-RLS-SS is a self-administered assessment tool recommended for children at least 9 years of age. Due to limited cognitive and reading abilities, younger children may need assistance completing the P-RLS-SS. The separate complementary parent questionnaire should be completed.

The next phase in development of the P-RLS-SS is the validation study. As developed, the plan is to field test the scale in order to determine the appropriateness of the questions and then to come up with a pared down final version of the scale with perhaps the addition of altered or additional questions as part of the validation procedure. The P-RLS-SS is the property of the International Restless Legs Syndrome Study Group (IRLSSG) and the IRLSSG web site can be consulted regarding its use http://www.irlssg.org.

#### Rating the Four Restless Legs Syndrome Diagnostic Criteria in Children

In one study in a modification of the P-RLS-SS the four primary criteria for the diagnosis of RLS were rated from least severe to most severe on a 0–4 scale in children 5–18 years of age as the sole measure of severity (27). The 5^th^ criterion, the exclusion of mimics, was part of the questionnaire as was the necessity for the child to be able to describe the symptoms in their own words. However. these items were not given a numerical score. A score of at least one on each of the four primary items and an affirmative answer on the mimics question and the child volubility question was necessary before the scale could be administered. In other words, the child had to meet the criteria for definite RLS before the symptoms could be rated. Severity thus ranges from 4 to 16. This approach has the advantage that it is short and easy to administer and the questionnaire serves both as a diagnostic tool and a severity rating tool. The disadvantage of the tool is that the four criteria are taken directly from material written for physicians and thus might not be understandable to either adults or children unless an examiner is personally present to verbally explain the questions to the subjects. It is also assumed that for very young children that the parent needs to be present to help with the responses. This approach holds promise but has also not yet been validated (27).

### Quality of Life Scales for Adult Restless Legs Syndrome

#### General Restless Legs Syndrome Quality of Life Scales

##### Kohen Restless Legs Syndrome Quality of Life Instrument

The questionnaire consists of 12 items that explore four different realms: firstly—consequences of RLS symptoms on sleep, activities of daily living, mood, and social interactions; secondly, everyday life, tiredness, and mood: thirdly pain and side effects of RLS medications; fourthly, behaviors to cope with RLS (28). A 6 point Likert scale is used to determine the severity for each question. The questionnaire has been validated recently in adults and the Kohnen RLS QOL index correlates well with the IRLS which also contains quality of life elements (r= 0.68), and to the Clinical Global Impression (r = 0.42), and to varying degrees to four of the RLS-6 domains (r = 0.33 to 0.57). To our knowledge the Kohnen RLS QOL instrument has not been utilized in children. The European Restless Legs Syndrome Study Group (EURLSSG) owns intellectual property rights over the Kohnen RLS-Qol with the Mapi Research Trust assigned the management of the instrument licenses and permission to use. For use please contact the Mapi Research Trust at e-mail Pro-information@mapi-trust.org or through website https://eprovide.mapi-trust.org/about/about-proqolid. The scale is available at a nominal cost to individual clinicians or investigators.

##### The Restless Legs Syndrome Quality of Life Questionnaire—Abetz

In this scale there are 18 administered questions including impact of RLS on the domains of daily life, emotional well-being, social life, and work life (29). Higher scores indicated higher quality of life. The scale has been validated in adults by comparison to the SF-36 and a modified version of the IRLS (IRLS—patient version or IRLS-PV). There was better correlation of the RLS QOL Questionnaire—Abetz with the SF-36 mental component summary (MCS) scale (r=0.5, P < .0001) than with the physical component summary (PCS) scale which was not significant. The RLS QOL Questionnaire—Abetz summary score was able to distinguish between those patients rated as mild, moderate or severe on the IRLS-PV (F= 52.22, P <0.0001). To our knowledge the RLS QOL questionnaire—Abetz has not been utilized in children.

##### The Restless Legs Syndrome Quality of Life Instrument

This is a self-administered scale with 17 questions (30). The 17 questions were analyzed by factor analysis and four factors were identified: Daily function, social function, sleep quality, and emotional well-being. The instrument has been validated in adults against related scales of the SF-36 (r=0.47 to 0.60) and related items of the IRLS (r= −0.45 to −0.77), but to our knowledge has not been utilized in children. The RLS-quality of life instrument (QLI) is owned and maintained by the Restless Legs Syndrome Foundation (RLSF), a nation-wide support group for RLS patients and their families. Permission to use the instrument, free of charge, must be sought from the RLSF http://www.rls.org/research.

### Restless Legs Syndrome Quality of Life Instruments Specific to Sleep

#### The Post-Sleep Questionnaire for Restless Legs Syndrome

This is a self-completed scale and tests five areas of interest over the last week: quality of sleep overall, ability to function in the day, frequency of RLS symptoms in the night, and RLS-related sleep disturbances and latency (31). The scale employs a Likert scale except for one open-ended question about the number of nights per week the patient has had RLS symptoms. Lower post-sleep questionnaire (PSQ) scores indicate worse sleep. The scale has been validated in adults against the IRLS, RLSQOL-Abetz, the Profiles of Moods States (POMS), and the Medical Outcomes Study (MOS)-Sleep Scale (p < 0.007 each) and also validated against the investigator and subject rated clinical global impression (CGI) (p < 0.0001). To our knowledge the PSQ has not been employed in children.

#### The Restless Legs Syndrome-Next Day Impact Questionnaire

There are 14 questions to determine the impact of RLS-related sleep loss on daily functioning (32). The questionnaire is designed to rate only a single day “today.” It is self-administered at night and the patients are asked to fill out the items based upon their recollection of the previous 12 h. The questions are rated on an 11 point scale with higher numbers indicating more daytime dysfunction from RLS-related sleep loss. The scale probes impairments in alertness, concentration, and mood. The questionnaire has demonstrated content validity in adults through interviews with RLS patients and development by RLS and measurement experts but it has not to our knowledge been employed in children.

#### The Subjective Post-Sleep Diary for Restless Legs Syndrome

This diary was developed to be answered after a single night of sleep in RLS patients in order to assess their nocturnal sleep the night before (33). The diary consists of 12 items that the patient answers, e.g., recall of the time that they went to bed, time they awakened for the final time, how long it took them to fall asleep initially, how long they were awake in the middle of the night, and how much of the time spent awake was estimated to be due to RLS symptoms. The degree to which sleep was restful and the quality of sleep are both rated on a 0–10 scale with higher scores representing better sleep. Eight items from the diary were each correlated with the total score of the IRLS, the patient global impression, and with five individual sleep constructs from the Medical Outcomes Study (MOS) sleep scale. The correlations varied greatly but were generally moderate to high. To our knowledge the subjective post-sleep diary (SPSD) has not been employed in children.

### General Quality of Life Scales Applied to Pediatric Restless Legs Syndrome

In our literature search we did not find any studies utilizing quality of life scales that were specifically designed for pediatric RLS and to our knowledge none of the validated quality of life scales for adult RLS have been used in children or adolescents. However, three separate studies utilized the general Peds Quality of Life Inventory (PedsQL) in RLS children and adolescents (19, 20, 34). The PedsQL has 23 items on a self-report scale for children 2–18 years of age that utilizes four subscales: 1) physical functioning; 2) emotional functioning; 3) social functioning; and 4) school functioning (19, 20, 34). In one of these studies sleep quality was evaluated by another general measure the 26 item Sleep Behavior Questionnaire (34). Each item was rated 1 (never) up to 5 (always) according to how often the specific sleep symptom occurred over the past 6 weeks with higher scores indicating more sleep problems and reduced sleep quality. Scores could range between 26 and 130. In another of these studies the Pediatric Symptoms Checklist (PSC) was used as a general measure of Quality of Life (19). In the study parents of the participants where asked to respond to 35 questions rating their child’s level of psychosocial problems. Responses to each question were made where never occurs = 0 points, occurs sometimes = 1 point, and occurs frequently = 2 points. The total score was then calculated.

## Discussion

This article is a review of tools for assessment of adult RLS, their applicability to children and of assessment tools specifically developed for childhood RLS.

### Diagnosis

Diagnostic criteria provide standards for research and clinical practice. Criteria for RLS in children have been developed and follow the adult criteria. A diagnosis of definite RLS requires children to describe symptoms in their own words. A single question for the diagnosis of RLS has been validated for adults and utilized in one epidemiology study of adolescents with RLS (10). The pediatric Emory RLS questionnaire has been developed as a diagnostic instrument for childhood RLS, has been utilized in two studies, but has yet to be validated (13, 14). A parental questionnaire, the RLSQ has been validated for the diagnosis of pediatric RLS but the full scale has not been published to our knowledge (15).

#### Severity

The IRLS (International Restless Legs Scale), the CGI (Clinical Global Impression), and the RLS-6 all of which have been validated for determining RLS severity in adults were administered without difficulty in one therapeutic study of adolescent RLS (17). In addition, the IRLS has also been utilized in another five studies of childhood and adolescent RLS (18–22). However, it is to be emphasized that the adult version of the IRLS has not been validated in children or adolescents. The pediatric Restless Legs Syndrome Severity Scale (P-RLS-SS) has been developed for use in children but has not been validated (5). A modification of the P-RLS-SS based upon rating the severity of the four diagnostic criteria for RLS has been developed for children but not yet validated (27).

#### Quality of Life and Sleep Scales

We could find no instances where validated adult scales for RLS quality of life or sleep had been utilized in children and we could find no Quality of Life Scales or sleep scales specifically developed for RLS children. However, three separate studies utilized the general Peds Quality of Life Inventory (PedsQL) in RLS children and adolescents (19, 20, 34) and one of these studies also employed the general Sleep Behavior Questionnaire (SBQ) as an additional measure of quality of life (34) Yet another of these studies also employed the pediatric symptom checklist (PSC) as another general measure of quality of life in children with RLS (19).

#### Summary

There is a need for the development and validation of RLS diagnostic instruments, severity scales and quality of life instruments that are specific to children. In the interim, the adult scales have been utilized successfully in adolescent RLS where language barriers are not a problem. If adult scales are to be used in younger children we recommend that the methodology employed by Furudate be employed, i.e., that the scale be administered “after a thorough discussion of symptoms and daytime functioning with the participants and their parents (19).” Based upon our clinical experience we would go further and suggest that this process occur not only after a discussion with the children and their parents but during the administration of the scale as well so that the process be as interactive as possible such that misunderstandings do not arise. Having the child and parent decide upon a joint response would be a further recommendation.

## Author Contributions

Both the authors PS and AW contributed equally to conceptualization and writing of the manuscript.

## Conflict of Interest

AW has received funding from the US National Institutes of Health (NIH) and from Mundipharma for studies on Restless Legs Syndrome (RLS) and is currently receiving funds from Arbor Pharmaceuticals for an industry sponsored study of adolescent RLS.

The remaining author declares that the research was conducted in the absence of any commercial or financial relationships that could be construed as a potential conflict of interest.
